# Viral and bacterial co-infection in severe pneumonia triggers innate immune responses and specifically enhances IP-10: a translational study

**DOI:** 10.1038/srep38532

**Published:** 2016-12-06

**Authors:** Jonathan Hoffmann, Daniela Machado, Olivier Terrier, Stephane Pouzol, Mélina Messaoudi, Wilma Basualdo, Emilio E Espínola, Rosa M. Guillen, Manuel Rosa-Calatrava, Valentina Picot, Thomas Bénet, Hubert Endtz, Graciela Russomando, Gláucia Paranhos-Baccalà

**Affiliations:** 1 Laboratoire des Pathogènes Émergents, Fondation Mérieux - CIRI - Inserm U1111, Lyon, France; 2 Virologie et Pathologie Humaine VirPath, CIRI - UCBL1 - Inserm U1111 - CNRS UMR 5308 - ENS de Lyon, Lyon, France; 3 Hospital General Pediátrico Niños de Acosta Ñu, Ministerio de Salud Pública y Bienestar Social, Paraguay; 4 Departamento de Biología Molecular y Biotecnologia, Instituto de Investigaciones en Ciencias de la Salud, Universidad Nacional de Asunción, Paraguay; 5 Infection Control and Epidemiology Unit, Edouard Herriot Hospital, Hospices Civils de Lyon, Lyon, France

## Abstract

Mixed viral and bacterial infections are widely described in community-acquired pneumonia; however, the clinical implications of co-infection on the associated immunopathology remain poorly studied. In this study, microRNA, mRNA and cytokine/chemokine secretion profiling were investigated for human monocyte-derived macrophages infected *in-vitro* with Influenza virus A/H1N1 and/or *Streptococcus pneumoniae.* We observed that the *in-vitro* co-infection synergistically increased interferon-γ-induced protein-10 (*CXCL10*, IP-10) expression compared to the singly-infected cells conditions. We demonstrated that endogenous miRNA-200a-3p, whose expression was synergistically induced following co-infection, indirectly regulates *CXCL10* expression by targeting suppressor of cytokine signaling-6 (*SOCS-6*), a well-known regulator of the JAK-STAT signaling pathway. Additionally, in a subsequent clinical pilot study, immunomodulators levels were evaluated in samples from 74 children (≤5 years-old) hospitalized with viral and/or bacterial community-acquired pneumonia. Clinically, among the 74 cases of pneumonia, patients with identified mixed-detection had significantly higher (3.6-fold) serum IP-10 levels than those with a single detection (*P* = 0.03), and were significantly associated with severe pneumonia *(P* < 0.01). This study demonstrates that viral and bacterial co-infection modulates the JAK-STAT signaling pathway and leads to exacerbated IP-10 expression, which could play a major role in the pathogenesis of pneumonia.

Community-acquired pneumonia (CAP) is a common inflammatory illness of the lungs that remains the major cause of pediatric mortality in low- and middle-income countries^1^. *Streptococcus pneumoniae* (SP) is the main causative agent of pneumonia; however, concomitant viral infection occurs in up to 66% of cases of CAP^2^,^3^. Clinical data suggest mixed infections are related to a higher severity of inflammatory disease^4^,^5^ - especially in secondary pneumococcal infection following influenza virus (IAV) infection^6^,^7^,^8^ - and mixed infection represents a relevant risk factor for pediatric intensive care hospitalization^9^. However, the mechanisms underlying the pathogenesis of mixed viral and bacterial infection remain poorly understood.

Assessment of induced blood immunomodulators during infection may assist clinical diagnosis and the management of severe CAP. Interferon (IFN)-γ-induced protein 10 (IP-10/CXCL10) appears to contribute to the pathogenesis of several diseases and has been suggested as a potential biomarker of viral infection^10^,^11^, late-onset bacterial infection in premature infants^12^, and a promising biomarker of sepsis and septic shock^13^,^14^. Combined analysis of IP-10 and IFN-γ has also been reported as a useful biomarker for diagnosis and monitoring therapeutic efficacy in patients with active tuberculosis^15^,^16^,^17^, and both remain detectable in the urine of patients with pulmonary diseases in the absence of renal dysfunction^18^.

With airway epithelial cells^19^, resident alveolar macrophages (AMs) and blood monocytes-derived macrophages (recruited into tissues under inflammatory conditions^20^,^21^) represent a major line of defense against both pneumococcal (through their high phagocytic capacity^22^,^23^,^24^) and influenza infection^25^,^26^. So far, no studies have yet focused on the intracellular mechanisms that regulate IP-10 in human blood leukocytes during mixed IAV and SP infection. Several studies indicated that host non-coding small RNAs (including microRNAs) may function as immunomodulators by regulating several pivotal intracellular processes, such as the innate immune response^27^ and antiviral activity^28^,^29^; both of these processes are closely related to toll-like receptor (TLR) signaling pathways.

In this study, we firstly investigated the *in vitro* intracellular mechanisms that mediate the innate immune response in IAV and/or SP infected human monocyte-derived macrophages (MDMs). Using this approach, we observed that mixed-infection of MDMs induces a synergistic production of IP-10 which can be related to a miRNA-200a/JAK-STAT/SOCS-6 regulatory pathway. Subsequently, in a retrospective analysis of clinical samples collected from children ≤5 years-old hospitalized with pneumonia, we confirmed that serum IP-10 level could be related to both viral and/or bacterial etiologies and disease severity.

## Results

### Characteristics of MDMs infected by IAV and/or SP

**Figure 1:**
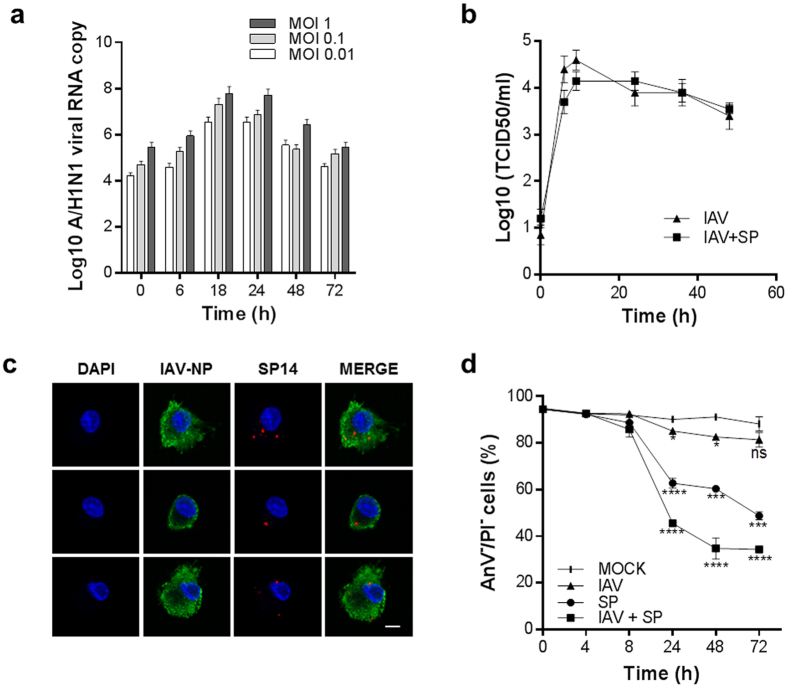
Single and mixed infection of human monocyte derived-macrophages (MDMs) by IAV and SP. (**a**) MDMs were infected with IAV at an MOI of 0.01, 0.1 or 1 and total RNA was extracted at 0, 6, 18, 24, 48 and 72 hours post-infection. The IAV A/H1N1 hemagglutinin (*HA*) gene was amplified by real-time qRT-PCR. (**b**) Influenza A/H1N1 viral load (TCID_50_) in the supernatant of single IAV-infected MDMs or IAV-infected MDMs following subsequent challenge with SP. (**c**) Confocal imaging of IAV and SP in mixed-infected MDMs at 8 hours post-IAV infection and 4 hours post-SP infection. DAPI, nuclear stain, blue. IAV, Influenza A virus nucleoprotein stain, green. SP, *S. pneumoniae* stain, red. Scale bar = 5 μM. (**d**) Impact of single and mixed IAV and SP infection on MDM cell viability. Statistical analyses were performed using two-way ANOVA with Tukey’s post-hoc test; *P < 0.05; **P < 0.01; ***P < 0.001; ****P < 0.0001.

Initially, we investigated *in vitro* the impact of single and mixed IAV and SP infection on MDMs. Firstly, active replication of IAV was assessed by qRT-PCR and quantification of new infectious viral particles in the cell supernatants (Fig. 1a,b). IAV titer increased over time after single infection with IAV and correlated with increased production of negative-strand IAV RNA. Maximum viral replication was observed at 18–24 hours post-infection, after which time both RNA replication and the quantity of infectious particles decreased. In this *in vitro* model, subsequent challenge of IAV-infected MDMs with SP had no significant impact on the production of new infectious viral particles (Fig. 1b). Together, these results indicate permissive and productive infection of MDMs by IAV. Secondly, we evaluated whether MDMs are permissive for both IAV and SP infection. The presence of pneumococci within IAV- and SP-infected primary MDMs was confirmed at 8 h post-infection (Fig. 1c), suggesting that MDMs are permissive for viral and bacterial co-infection in the early steps of infection. Importantly, confocal co-detection of mixed IAV and SP was only effective following 8 h post-infection due to the bactericidal impact of SP internalization within human macrophages (after 24 h, data not shown). Thirdly, we evaluated the impact of single and mixed infection with IAV and SP on MDM viability. Mixed infection significantly decreased cell viability (65.2 ± 4.5% total cell death at 48 hours post-infection; *P* < 0.0001) compared to single SP and IAV infection (39.6 ± 1.7% and 17.4 ± 1.1% total cell death, respectively; Fig. 1d). Taken together, these results confirmed human MDMs are permissive to mixed viral and bacterial infection.

### mRNA, microRNA and protein expression profiling reveal an overall induction of the host innate immune response following IAV and/or SP infection of MDMs

**Figure 2:**
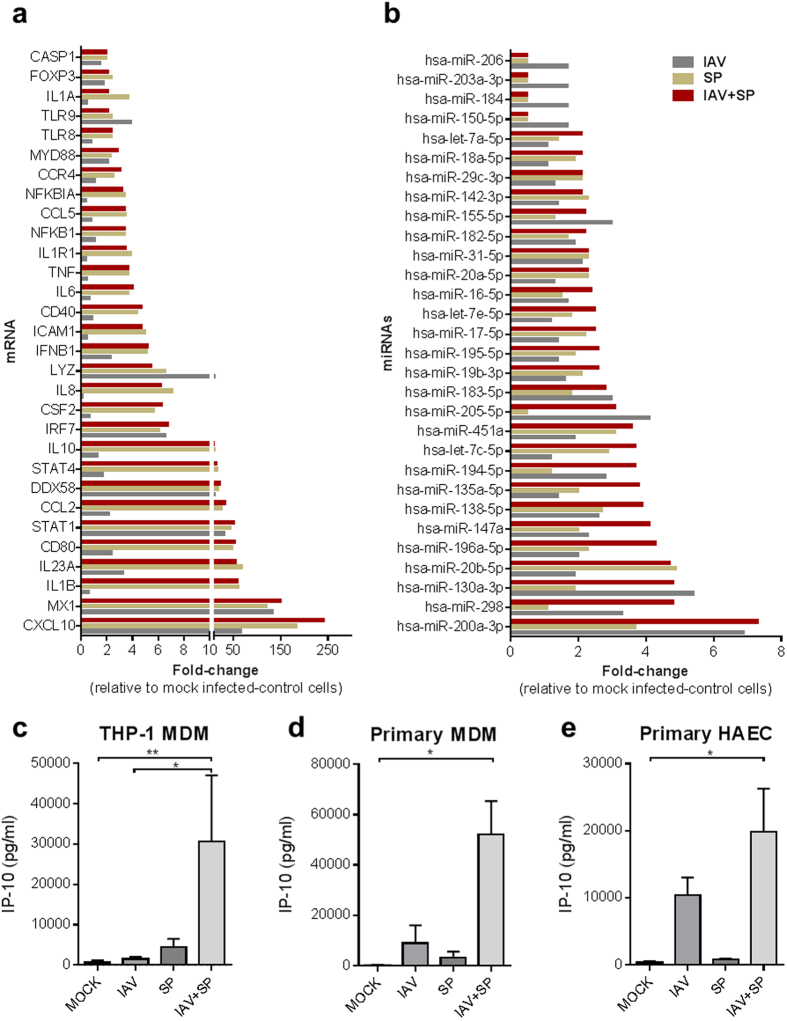
Investigation of host mRNA, miRNA and protein expression levels during IAV and/or SP infection of MDMs or HAEC. (**a,b,c**) THP-1 MDMs were infected with IAV (MOI = 1) for 4 h before SP (MOI = 1) was added. Total cellular mRNA (**a**) or miRNA (**b**) were extracted 24 h post-infection, reverse-transcribed and amplified using specific RT^2^ profiler PCR Arrays. Major differentially expressed mRNA or miRNAs are shown. (**c**–**e**) THP-1 MDMs, primary human MDMs or primary airway epithelial cells (HAEC) were infected with IAV (MOI = 1) for 4 h before SP (MOI = 1) was added. The concentration of IP-10 in cell supernatant (24 h post-infection) was determined by a multiplex immunoassay on a FlexMap3D Luminex platform. Values represent mean ± SEM of four (**c**) biological replicates, four (**d**) independent experiments with different donors, three (**e**) biological replicates from one donor. Statistical analyses for each panel of experiments (**c–e**) were performed using a Kruskal-Wallis test (non-parametric, one-way ANOVA with Dunn’s post-hoc test); *P < 0.05; **P < 0.01.

To investigate the innate immune response orchestrated by IAV- and SP-infected human MDMs, we firstly evaluated the expression of 84 genes involved in the innate and adaptive immune responses (Table S1); the major differentially-expressed genes are summarized in Fig. 2a. Expression profiling indicated an overall induction of genes related to the JAK-STAT, NF-Κβ and TLR signaling pathways. Indeed, all interferon-stimulated genes (ISGs) screened, including *CXCL10* (fold-change [FC] = 240.9), *CCL-2* (FC = 34.2) and *MX-1* (FC = 151.4) were upregulated following mixed infection compared to uninfected cells, most of which are closely related to *STAT-1* (FC = 52.3), *IRF-7* (FC = 6.8) and *IFNB1* (FC = 5.2) also found upregulated in mixed infected cells. Secondly, we investigated the endogenous microRNA expression profiles of IAV- and SP-infected MDMs. A selection of microRNAs that were found to be differentially-expressed under different infection conditions are shown in Fig. 2b and Table S2. MiRNA-200a-3p was overexpressed after both single IAV (FC = 6.9), single SP (FC = 3.7) and mixed IAV/SP infection (FC = 7.3), indicating this miRNA may play a role in the innate immune response to viral and bacterial co-infection. Similar miRNA-200a-3p dysregulation profiles were obtained following IAV and/or SP infections of human macrophages-like (THP-1 monocytes-derived macrophages) or primary MDMs (**data not shown**). Thirdly, the secreted levels of various antiviral, pro-inflammatory and immunomodulatory cytokines/chemokines were assayed in IAV- and SP-infected- THP-1 and primary MDM cell supernatants. We observed a remarkable correlation between the mRNA and protein expression profiles of single or mixed infected MDMs especially regarding CXCL-10 and IP-10 expression. Indeed, the level of IP-10 was synergistically increased in the supernatant of IAV-infected THP-1 MDMs exposed to SP (mean: 30,589 ± 16,484 pg ml^−1^) compared to single IAV infection (1,439 ± 566.5 pg ml^−1^) and single SP infection (4,472 ± 2,001 pg ml^−1^; P≤0.05; Fig. 2c) at 24 hours after infection. In those cells, IP-10 expression reduced over time (48 to 72 hours), coinciding with a significant higher proportion of necrotic and apoptotic cells (Fig. 1d). The synergistic expression of IP-10 was similarly observed at 24 hours post-infection using primary MDMs (Fig. 2d). Significantly increased secretion of the other tested cytokines and chemokines was not observed post-infection, even in mixed infected MDMs (Fig. S1). Interestingly, a significant production of IP-10 was also observed in supernatants of primary human airway epithelial cells (HAEC) mixed-infected by IAV and SP compared to the single infections (Fig. 2e). Taken together, the mRNA and protein profiling results suggested that mixed viral and bacterial infection of MDMs induces a synergistic pro-inflammatory response related to the type-1 interferon and JAK-STAT signaling pathways, with IP-10 as signature of IAV/SP co-infection. Among all microRNAs screened, miR-200a-3p was the most overexpressed in IAV/SP co-infection of human MDMs. In the remainder of this study, we decided to investigate the interconnection between miR-200a-3p expression and the innate immune response.

### Endogenous miRNA-200a-3p expression correlates with CXCL10 (IP-10) induction following mixed IAV and SP infection of human MDMs

**Figure 3:**
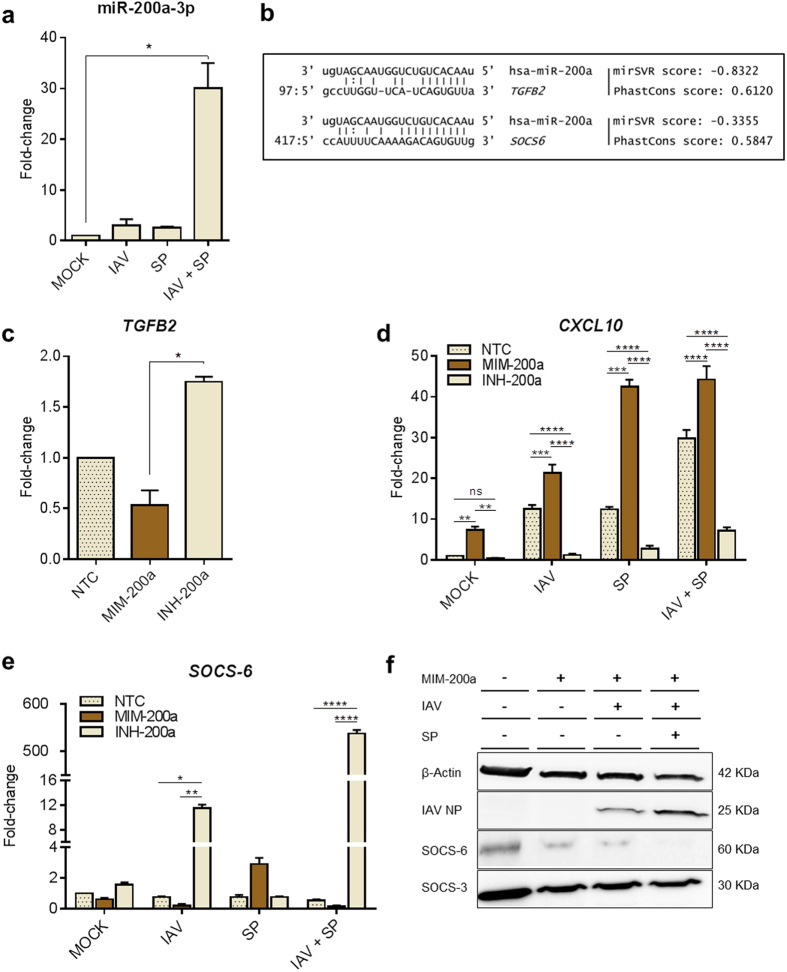
Functional analysis of the synergistic induction of miR-200a-3p in MDMs after mixed infection with IAV and SP. (**a**) Preceding IAV infection elevates miR-200a-3p expression after mixed infection with SP. MDMs were infected with IAV for 4 hours, then infected with SP, incubated for 20 h and total endogenous miRNAs were purified. A specific PCR-probe assay targeting miR-200a-3p was used to assess the fold changes in miR-200a-3p expression in mock-transfected and infected cells. (**b**) *In silico* predictive target alignment showing that miR-200a-3p targets the 3′ UTRs of both *TGFB2* and *SOCS6*. (**c**) *TGFB2*, (**d**) *CXCL10* and (**e**) *SOCS6* expression profiles in MDMs transfected with negative transfection control (NTC), miR-200a mimic (MIM-200a) or miR-200a inhibitor (INH-200a). At 18 h after transfection, the MDMs were singly or mixed infected as described previously. At 8 h post-IAV and/or SP infection, total mRNA was extracted and amplified by PCR using specific primers for the indicated genes. Values represent median ± IQR (**a, c**) or mean ± SEM (**d, e**) of three biological replicates. Statistical analyses were performed using a Kruskal-Wallis test (non-parametric, one-way ANOVA with Dunn’s post-hoc test) for data presented in (**a, c**). An ordinary two-way ANOVA (with Tukey’s post-hoc multiple comparison test) was used for data presented in (**d, e**). *P < 0.05; **P < 0.01; ***P < 0.001; ****P < 0.0001. (**f**) Western blot analysis of SOCS-6, SOCS-3, IAV nucleoprotein (NP) and β-actin expression in MDMs transfected with negative transfection control (NTC), miR-200a mimic (MIM-200a) or miR-200a inhibitor (INH-200a), cultured for 18 h, and then infected as described previously. Cell lysates were harvested 24 hours post-infection.

Using a specific Taqman probe assay targeting miR-200a-3p, we confirmed a significant upregulation of miR-200a-3p following mixed IAV and SP infection of human MDMs (Fig. 3a). In this experiment, a more marked up-regulation of miR-200a-3p was observed following IAV+SP compared to results obtained previously (Fig. 2b). This discrepancy has been attributed to the use of two different approaches to quantify miR-200a-3p expression. The use of a target-specific stem-loop reverse transcription primer in Fig. 3a allows a better sensitivity of miR-200a-3p detection compared to the non-specific fluorescent dye used in Fig. 2b. As the general trend was suggestive of a synergistic induction of miR-200a-3p in response to mixed infection (Fig. 3a), we hypothesized microRNA-200a-3p may play a role in the regulation of *CXCL10* (IP-10), which was also synergistically upregulated in mixed-infected MDMs (Fig. 2c and d) and primary HAEC (Fig. 2e). To test this hypothesis, we investigated the effects of overexpressing (MIM-200a) or inhibiting (INH-200a) microRNA-200a in MDMs. As *TGFB2* is known to be targeted by miR-200a-3p (Fig. 3b, *in silico* alignment), we monitored its expression to assess the efficiency of mimicking/inhibiting miR-200a-3p in THP-1 MDMs. As expected, *TGFB2* mRNA was significantly downregulated in MIM-200a-transfected MDMs (FC = 0.57) and upregulated in INH-200a-transfected MDMs (FC = 1.70), compared to mock-transfected cells (Fig. 3c). Overexpressing miR-200a-3p (MIM-200a) in single and mixed IAV/SP-infected cells enhanced the levels of *CXCL10* at 24 h post-infection, whereas partial inhibition (INH-200a) downregulated the expression of *CXCL10* (Fig. 3d). These results suggested miR-200a-3p indirectly regulates *CXCL10* and led us to hypothesize that miR-200a-3p controls a potential repressor of the JAK-STAT signaling pathway.

### MiRNA-200a-3p indirectly regulates IP-10 expression by targeting SOCS6

As shown in Fig. 2a, several JAK-STAT signaling pathway genes were deregulated in mixed IAV- and SP-infected human MDMs; therefore, we hypothesized that miR-200a-3p directly regulates a regulator of the JAK-STAT signaling pathway. Predictive target analysis indicated that the 3‘ UTR of suppressor of cytokine signaling-6 (*SOCS6*) may be targeted by miR-200a-3p (Fig. 3b). SOCS proteins constitute a class of negative regulators of JAK-STAT signaling pathways that are induced by both cytokines and TLR signaling. MiRNA-200a-3p was not predicted to target any of the other six members of the SOCS gene family. Transfection of human MDMs with MIM-200a downregulated *SOCS6* (FC = 0.57) while inhibition of miR-200a-3p (INH-200a) upregulated *SOCS6* (FC = 1.55), confirming that miR-200a-3p effectively regulates the expression of *SOCS6* (Fig. 3e). Moreover, *SOCS6* was synergistically downregulated in IAV- or IAV/SP-infected MDMs overexpressing miRNA-200a (Fig. 3e), suggesting that both infection and miR-200a-3p negatively regulate the expression of *SOCS6*. Finally, western blotting confirmed that expression of SOCS-6 sharply reduced following infection, especially after mixed IAV and SP infection (Fig. 3f). These results indicate miR-200a-3p is strongly induced in response to mixed viral and bacterial co-infection, which in turn leads to downregulation of the JAK-STAT regulator SOCS-6 at both the mRNA and protein levels and subsequent upregulation of IP-10.

### Clinical investigation of innate immune response related to pneumonia etiology

The *in vitro* analyses demonstrated mixed IAV and SP infection of human MDMs and HAEC induced significant production of IP-10. As blood leukocytes and respiratory tract epithelial cells actively contribute to inflammation during pneumonia, we hypothesized the level of IP-10 in serum of patient with pneumonia may be both indicative of mixed respiratory infection and disease severity. As part of a prospective, hospital-based, multicenter case-control study on the etiology of pneumonia among children under 5-years-old, a total of 74 patients (44 male, 30 female) were included in this pilot evaluation. According to WHO guidelines, retrospective analysis indicated 44 (59.5%) children had clinical signs of non-severe pneumonia and 30 (40.5%) children had signs of severe pneumonia. The main patient characteristics at inclusion are shown in Table 1. Patients with severe pneumonia had significant more recorded episodes of dyspnea (P < 0.001), cyanosis (P = 0.03), lower chest indrawing (P < 0.001), dullness to percussion (P < 0.001) and lethargy (P < 0.001) during chest examination than patient with non-severe pneumonia. Moreover, pleural effusions were significantly more observed among critically ill patients and the duration of hospitalization was significantly longer for the children with severe pneumonia than for those with non-severe pneumonia (*P* = 0.0015). Two deaths occurred within the group of children retrospectively defined with severe pneumonia. Evaluation of the systemic inflammatory response of the 74 cases is shown in Table 2. Serum level of CRP, IP-10, PCT, G-CSF, IL-6, IL-8 and MIP-1β were significantly more elevated in serum samples from critically ill patients. Patients with severe pneumonia had significantly higher (4.2-fold) serum IP-10 levels than those with a non-severe pneumonia (*P* < 0.001) suggesting IP-10 as a promising prognostic marker in pneumonia. Diagnostic accuracy measures for predicting pneumonia severity using blood-based biomarkers are summarized in Table S3. Briefly, in this study, the optimal IP-10 cut-off value for identifying patient with severe pneumonia was 4,240 pg ml^−1^, with an area under the receiver operating characteristic curve of 0.69 (95% CI, 0.57 to 0.82, P < 0.001). Defining as positive a serum IP-10 level above this cut-off resulted in a sensitivity of 63.3%, specificity of 63.6% and a positive likelihood ratio of 1.74. Prognostic values of IP-10 were closed to procalcitonin (PCT; AUC = 0.70; 95% IC, 0.58 to 0.82, *P* < 0.001) and IL-6 (AUC = 0.70; 95% IC, 0.58–0.83, *P* < 0.001).

### IP-10 is significantly associated with viral and bacterial co-detection and pneumonia severity

Multiplex PCR-based screening of respiratory and blood samples reveal a high variety of pathogen associations (Table 3). Respiratory viruses were detected in the nasal aspirates (NAs) of 63/74 patients (85.1%). Etiological bacteria of pneumonia (*S. pneumoniae*, *n* = 19; *S. aureus*, *n* = 1; or *H. influenzae* type B, *n* = 7) were identified via real-time PCR in the blood samples of 27/74 (36.5%) of the patients. Multiplex PCR assays allowed the identification of respiratory bacteria in the blood of 19 patients with negative blood culture results. Among the 74 cases PCR-positive for respiratory pathogens, a single virus or bacteria were detected in the NAs of 7 (9.4%) and 3 (4.0%) patients, respectively; these 10/74 (13.5%) cases were defined as the single infection group. The mixed infection group included the 62/74 (83.8%) cases in which (1) multiple viruses and/or bacteria were identified in NAs (38/74; 51.3%) without any bacteria identified in blood samples or (2) one or more viruses and/or bacteria were identified in NAs and associated with a blood bacteremia (24/74; 32.4%). We evaluated whether IP-10 serum level could correlate with the viral and bacterial etiologies of pneumonia. Patients with mixed infection had significant higher (3.6-fold) IP-10 serum level than patient with single detection (*P* *=* *0.03*; Table 4). A stratified analysis reveals that the highest IP-10 serum level was observed among patients with both several respiratory pathogens identified (mixed-detection group) and severe pneumonia (14,427 pg ml^−1^, IQR (3,981–82,994). In detail, a remarkable IP-10 serum level (142,531 pg ml^−1^), representing 33-fold higher above cut-off value predicting pneumonia severity was observed in patient with hRV in NA co-detected with *S. pneumoniae* (serotype 14) in pleural effusion and blood. In concordance with our *in-vitro* model of co-infection, a significant IP-10 level (90,338 pg ml^−1^) was quantified in blood sample of patient with severe bacteremic pneumococcal (serotype 14) pneumonia with a positive co-detection of Influenza B virus in NA. Taken together, these results suggest that high serum IP-10 levels are significantly associated with mixed viral and bacterial detection and also related to pneumonia pathogenesis.

## Discussion

**Figure 4:**
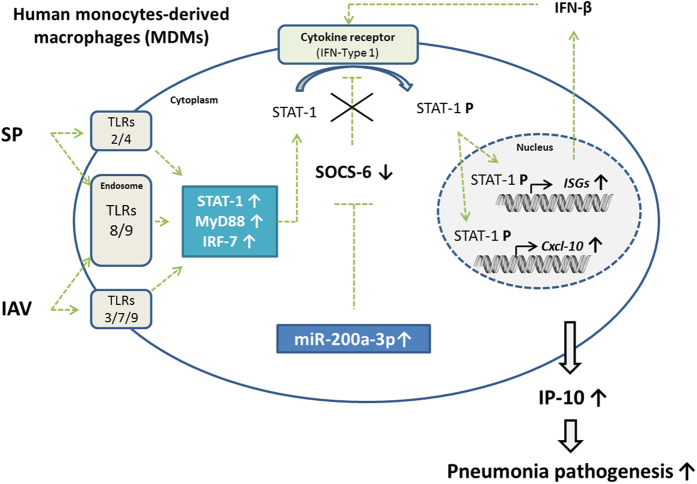
IAV and SP co-infection of MDMs exacerbates induction of IP-10: putative role for miR-200a-3p in regulating the JAK-STAT signaling pathway. Both the *in vitro* and clinical studies indicated IP-10 is associated with mixed viral/bacterial infection in pediatric community-acquired pneumonia. *In vitro*, miR-200a-3p was synergistically induced following mixed IAV/SP infection of human MDMs and found to indirectly regulate *CXCL10* (IP-10) expression by targeting the suppressor of cytokine signaling-6 gene (*SOCS6*), a well-known repressor of the JAK-STAT signaling pathway. *In vivo*, IP-10 was significantly elevated in the serum samples of pediatric patients with mixed viral/bacterial severe pneumonia compared to patients with single detection (*P* = 0.03) and non-severe pneumonia (P < 0.01).

This study provides additional *in vitro* and clinical data to improve our understanding of the immunopathology of mixed viral and bacterial pneumonia (Fig. 4).

The *in vitro* model of influenza and pneumococcal superinfection of human MDMs demonstrated that mixed infection synergistically induced release of the pro-inflammatory chemokine IP-10, strongly suggesting human blood leukocytes contribute to the immunopathology of pneumonia. Additionally, transcriptomics and omics analyses provided new data on the inflammatory pathways that are activated during mixed infection and related to synergistic induction of the pro-inflammatory chemokine IP-10 in mixed infected cells. Our observations are consistent with a recent study describing IP-10 induction as host-proteome signature of both viral and bacterial infections^30^. Of the differentially-expressed genes observed in mixed infected MDMs, the transcription factors *STAT-1* and *IRF-7* appear to play crucial roles in the regulation of interferon-stimulated genes including *CXCL10* (IP-10). By focusing on the intracellular mechanisms that regulate inflammatory pathways, we demonstrated a novel role for miRNA-200a-3p in the regulation of *CXCL10* (IP-10). These observations are consistent with previous reports showing that RNA virus infection upregulates miR-155 in macrophages and dendritic cells and also regulates suppressor of cytokine signaling 1 (*SOCS1*), suggesting the existence of a miRNA/JAK-STAT/SOCS regulatory pathway during viral infection^29^. Our study suggests co-infection leads to overexpression of miR-200a-3p, which in turn targets and downregulates the JAK-STAT regulator *SOCS-6* and consequently increases *CXCL10* (IP-10) expression. Interestingly, a complementary *in-silico* approach reveals that several microRNAs that were found dysregulated in our experiments of IAV and SP co-infection of MDMs or HAEC, might target several genes of SOCS family and play similar role than miR-200a-3p. Indeed, miRNA-142-3p might target *SOCS4, 5, 6* mRNA while miRNA-194-5p might target *SOCS2, 3, 4, 5* and *7* mRNA. These observations underline that intra-cellular regulation of IP-10 is not limited to the contribution of a sole microRNA. A complex inter-relationship between numerous host microRNAs and inhibitors of the JAK-STAT signaling pathway occur to control host innate inflammatory response against viral and/or bacterial infections.

Clinically, the majority of pediatric CAP cases in this study were associated with both positive viral and/or bacterial detection. Respiratory microorganisms were detected in 97% of cases; 51.3% of which were viral-viral, viral-bacterial or bacterial-bacterial co-detected only in nasal aspirates, 32.4% of which co-detected in both nasal aspirates and blood samples. These data are consistent with previous etiological studies of pediatric CAP^3^,^31^,^32^,^33^. *S. pneumoniae* was the major bacteria identified in blood (19/74; 25.7%) and mainly co-detected with respiratory viruses in NAs (16/19; 84.2%). We observed a very high diversity of viral and bacterial associations in biological samples from children with pneumonia. In comparison with IAV and SP14 combination evaluated *in-vitro*, no pneumonia cases were singly influenza and pneumococcus infected, and no similar co-detection with those two pathogens has been clinically observed. Nevertheless, Influenza B (IVB) virus was identified in 5 patients and two of them had a positive SP co-detection in blood (one non-typable strain and one serotype 14 using our molecular typing test). IVB and SP14 combination seems to be the nearest pathogen co-detection to that *in-vitro* investigated. Clinically, this co-detection was associated with both a very high IP-10 expression and a very severe pneumonia case definition. Interestingly, our translational pilot evaluation reveals IP-10 expression can be induced by several different viral and/or bacterial combinations. As immune response to each pathogen is different, further *in-vitro* investigations using different pathogens associations are needed to better characterize the mechanisms involved in the immunopathology of pneumonia.

In this cohort, highest serum IP-10 levels were identified among patients with both several pathogen detected and severe pneumonia, suggesting a significant role of IP-10 on pneumonia pathogenesis. Indeed, high plasma levels of IP-10 have previously been reported in patients with sepsis^12^, and were associated with high mortality rate, especially among patients with CAP^34^. Additionally, the IP-10-CXCR3 axis has been related to acute immune lung injury and lymphocyte apoptosis during the development of severe acute respiratory syndrome (SARS)^35^,^36^. Moreover, an *in vivo* study that modeled influenza and pneumococcal superinfection in mice indicated that pro-inflammatory chemokines, including IP-10, play a crucial role in influenza-induced susceptibility to lung neutrophilia, severe immunopathology and mortality^37^. In this study, markedly elevated IP-10 (92,809 pg ml^−1^) combined with the highest PCT level (74.4 pg ml^−1^) were quantified in the serum sample of a child who died, in whom *S. pneumoniae* (serotype 9 V) was identified in the blood (PCR and blood culture) and co-detected with *Haemophilus influenzae* type B in nasal aspirate. These observations suggest an interrelationship between co-detection, elevated serum IP-10 and the pathogenesis of pneumonia.

Several limitations of this pilot translational study need to be acknowledged before concluding mixed infection is related to elevated IP-10 and disease severity. Indeed, although viral shedding (e.g., of HRV and HBoV) is common in asymptomatic children, we were unable to evaluate the levels of immunomodulators in the serum samples of a control group. Moreover, although the samples were collected within the first 24 hours after admission, only a single blood sample was processed for each patient. Therefore, a larger, longitudinal study on the etiology and severity of pneumonia will be necessary to confirm these results. In conclusion, the present findings suggest that mixed respiratory infections and IP-10 may play major, interconnected roles in the pathogenesis of pneumonia. Clinically, assessment and monitoring of induced IP-10 serum level may assist clinicians to improve diagnosis and patient management of severe community-acquired pneumonia.

## Materials and Methods

### Viral and bacterial strains

The seasonal influenza A/H1N1 virus (IAV; A/Solomon Islands/03/06) was obtained from the National Influenza Reference Center (Lyon, France). Encapsulated *Streptococcus pneumoniae* serotype 14 (SP) was obtained from the National Reference Center for Streptococci, Department of Medical Microbiology, University Hospital, Aachen, Germany.

### Cell culture conditions

Peripheral blood mononuclear cells (PBMCs) were isolated from whole blood of four healthy donors (female/male ratio: 1:3; range of age: 21–65 years) using Ficoll-Paque plus (GE Healthcare Life Sciences, Little Chalfont, United Kingdom) and monocytes were purified using magnetic CD14 MicroBeads (Miltenyi Biotec, Bergisch Gladbach, Germany). The purity of isolated CD14+ cells was consistently > 90%, as determined by flow cytometry (Accuri C6; BD Biosciences, San Jose, California, USA). Primary MDMs were obtained after 6 days of differentiation in RPMI-1640 glutamax supplemented with 10% heat-inactivated FBS and 10 ng ml^−1^ M-CSF (Miltenyi Biotec). THP−1 MDMs were obtained by culturing cells with 10 ng ml^-1^ phorbol myristate acetate (PMA; Invivogen, Toulouse, France) for 72 hours. Human airway epithelial cells (HAEC, bronchial cell type) originated from a 54-years old woman with no pathology reported (batch number MD056501) were provided by Mucilair (Epithelix, Geneva, Switzerland). Sterility, tissue integrity (TEER), mucus production and cilia beating frequency have been certified by the company.

### Multiplex real-time qRT-PCR co-detection of IAV and SP

Multiplex real-time qRT-PCR was performed to quantify IAV (*H1* hemagglutinin gene, provided by Pr AD Osterhaus from the Department of Virology, Erasmus Medical Center, 3015 GE Rotterdam, Netherland) and SP (autolysin A gene, *LytA,* unpublished home-made primers and probe design) in MDMs. The primers and probes (*H1-*F 5′-GAATAGCCCCACTACAATTGGGTAA-3′; *H1*-R 5′-GTAATTCGCATTCTGGGTTTCCT-3′; *H1*-P 5′-FAM-AAGATCCATCCGGCAACGCTGCA-BHQ1-3′ and *LytA*-F 5′-GCGGAAAGACCCAGAATTAG-3′; *LytA*-R 5′-GAATGGCTTTCAATCAGTTCAAC-3′; *LytA*-P 5′-Cy5-TCTcAGcATtCCaACCGC-BHQ-2-3′) were multiplexed using the AgPath-ID™ One-Step RT-PCR kit (Life Technologies; Carlsbad, California, USA) according to the manufacturer′s instructions; the cycling program was adjusted to 50 °C for 15 minutes; 95 °C for 10 minutes; 40 cycles of 8 seconds at 95 °C (denaturation) and 34 seconds at 50 °C with fluorescence detection (annealing and extension). Absolute quantification of viral hemagglutinin and pneumococcal *LytA* copy number were calculated using the cycle threshold (Ct) values and in-house standard curves of A/H1N1 influenza virus and *S. pneumoniae* serotype 14.

### Co-detection of IAV and SP by immunofluorescence

Single or mixed infected cells were washed with PBS, fixed with 4% paraformaldehyde for 20 minutes at room temperature, permeabilized in PBS-0.1% Triton X-100, and incubated with PBS containing 1% BSA and 3% human serum to block non-specific binding. Mouse monoclonal anti-Influenza A Virus Nucleoprotein (#ab20921; Abcam, Cambridge, United Kingdom) and goat anti-mouse IgG H&L (AF-488, #A11029; Life Technologies) were used to detect IAV. Rabbit polyclonal anti-*Streptococcus pneumoniae* antibody (#ab20429; Abcam) and goat anti-rabbit IgG H&L (AF-647, #ab150083; Abcam) were used to detect SP. Cells were imaged using a Leica DMI 3000B microscope equipped for fluorescence imaging.

### Gene expression profiling

Total cellular mRNA was purified using the RNeasy kit (Qiagen, Hilden, Germany). Reverse-transcription of total mRNA was performed using the RT^2^ First Strand Kit (SABiosciences, Hilden, Germany). The expression of 84 genes involved in the human innate and adaptive immune responses was evaluated using the RT^2^ profiler™ PCR Array (SABiosciences) according to the manufacturer’s recommendations. The ΔΔCt method was applied to calculate the fold changes in gene expression for each gene relative to uninfected control cells using the web-based RT^2^ profiler PCR Array Data Analysis software (SABiosciences).

### MicroRNA profiling array

Total cellular microRNAs were purified using the miRNeasy Mini kit (Qiagen) and reverse-transcribed using the miScript Reverse Transcription kit (Qiagen). The profiling of 84 miRNAs was performed using the Human Immunopathology miScript miRNA PCR Array kit (Qiagen) according to the manufacturer’s instructions. Data were analyzed using the miScript miRNA PCR array data analysis web portal.

### In silico miRNA target prediction

MiRNA target genes were retrieved and compiled using TargetScan^38^ and microRNA.org resource^39^. The interactions between miRNAs and intracellular pathways were predicted using DIANA-miRPath v2.0^40^.

### Monocyte-derived macrophage (MDM) co-infection model

Human primary CD14+ or THP-1 MDMs were seeded in 24-well plates (0.5 × 10^6^ per well) in triplicate, exposed to Influenza A H1N1 (A/Solomon islands/3/2006) virus (IAV) under serum-free conditions for 1 hour and then cultured for 3 hours in fresh RPMI-1640 containing 2% FBS. *Streptococcus pneumoniae* (SP) serotype 14 was added at 4 hours after IAV infection. Gentamicin (10 μg ml^−1^) was added 2 hours after SP infection (i.e. 6 hours post-influenza infection) and maintained in the culture media throughout the experiment to kill extracellular bacteria and limit bacterial growth. Cell viability was determined by flow-cytometry using the FITC/Annexin V apoptosis detection kit (BD Biosciences), according to the manufacturer’s instructions.

### Functional analysis of miR-200a-3p

For miR-200a-3p inhibition/mimic assays, 1 × 10^6^ THP-1 MDMs were transfected with either 30 nmol *miR*Vana® hsa-miR-200a-3p inhibitor (#4464084), hsa-miR-200a-3p mimic (#4464066), miRNA Inhibitor Negative Control #1 (#4464077) or miRNA Mimic Negative Control #1 (#4464058; all Life Technologies) in OptiMEM (Life Technologies) using 3 μl Invitrogen RNAiMAX Lipofectamine/well in 12-well plates. After 18 hours, cells were washed and infected as previously described. The efficiency of the miR-200a-3p inhibitor/mimic was evaluated 24 hours after mixed infection by purifying total miRNAs (*mir*Vana® miRNA Isolation Kit, Ambion, Life Technologies), reverse transcription and specific amplification of miR-200a-3p using the TaqMan MicroRNA Reverse Transcription kit (Life Technologies, #4366596) combined with a specific miRNA probe assay (Life Technologies, #4427975). In this assay, fold changes have been defined by the ΔΔCt method using control RNU-44 and -48 as reference microRNAs. Total mRNA was purified from transfected and infected MDMs using the RNeasy kit (Qiagen) and specific primers were used to amplify transforming growth factor beta-2 (*TGFB2*; F: 5′-CCATCCCGCCCACTTTCTAC-3′, R: 5′-AGCTCAATCCGTTGTTCAGGC-3′), *SOCS6* (F: 5′-AAGAATTCATCCCTTGGATTAGGTAAC-3′, R: 5′-CAGACTGGAGGTCGTGGAA-3′)^41^, *CXCL10* (IP-10; F: 5′-GTGGCATTCAAGGAGTACCTC-3′, R: 5′-TGATGGCCTTCGATTCTGGATT-3′) and β-Actin (F: 5′-CTCTTCCAGCCTTCCTTCCT-3′, R: 5′-AGCACTGTGTTGGCGTACAG-3′). Rabbit polyclonal antibodies against β-actin (# ab8227), SOCS-6 (#ab13950), SOCS-3 (#ab16030) and goat polyclonal anti-Influenza A/H1N1 (#ab119978, all Abcam) were used for Western-blotting.

### Multiplex immunoassay of inflammatory mediators

Serum samples and cell culture supernatants were screened for the presence of 27 human cytokines and chemokines using the Bio-Plex Pro Human Cytokine Standard 27-Plex kit (Bio-Rad, Hercules, California, USA) on a FLEXMAP 3D^®^ analyzer (Luminex, Austin, Texas, USA).

### Community-acquired pneumonia cohort

As part of a prospective, hospital-based, multicenter case-control study on the etiology of pneumonia among children under 5-years-old (study protocol published in^42^), a total of 74 patients (44 male, 30 female) admitted to the Hospital Pediátrico Niños de Acosta Ñu, San Lorenzo, Paraguay, between 2010–2013 were included in our study. Briefly, pneumonia cases were defined by the presence of 1) cough and/or dyspnea, and 2) tachypnea, as defined by the World Health Organization (WHO) (age <2 months: ≥60/minute; age 2–11 months: ≥50/minute; age 1–5 years: ≥40/minute)^43^, and 3) absence of wheezing at auscultation, and, 4) first symptoms appearing within the last 14 days, and 5) radiological confirmation of pneumonia as per WHO guidelines^44^. Based on these primary criteria defining pneumonia cases, all 74 cases were retrospectively re-evaluated according to the WHO “Pocket book of hospital care for children”^45^ criteria to evaluate pneumonia severity. Cases that died during the study, or who had at least one additional clinical signs including central cyanosis, dullness to percussion during chest examination, prostration/lethargy, pleural effusion observed on chest radiography were retrospectively included in the severe pneumonia group. Patients without any of these additional clinical signs were included in the non-severe pneumonia group.

### Clinical and molecular analysis

Nasopharyngeal aspirates (NAs) and whole blood samples were collected from children within 24 hours of admission. Whole blood samples were used for complete blood counts, blood culture and multiplex real-time PCR to identify *Staphylococcus aureus*, *Streptococcus pneumoniae* and *Haemophilus influenzae* type B^46^. *S. pneumoniae* serotypes were defined using a 11 multiplex real-time PCR assay targeting the 40 most frequently represented serotypes or serogroups according to protocol developed by Messaoudi *et al*.^47^. Serum C-reactive protein (CRP; AssayPro, St. Charles, Missouri, United States) and Procalcitonin (PCT; VIDAS *B.R.A.H.M.S*; bioMérieux) were quantified from whole-blood samples. Multiplex real-time non quantitative PCR (Fast-Track Diagnostic, Sliema, Malta) was used to detect 19 viruses and five bacteria in the respiratory specimens (NAs and pleural effusions). Mixed detection was defined as 1) PCR-positive for multiple viruses in NAs, 2) positive blood culture or PCR-positive for multiple bacteria in blood or 3) PCR-positive for one or multiple viruses in NAs and one or multiple bacteria in blood (identified by PCR and blood culture).

### Ethical approval

The study protocol, informed consent statement, clinical research form, any amendments and all other study documents were submitted to and approved by the Ethical Committee of the Instituto de Investigaciones en Ciencias de la Salud, the Universidad Nacional de Asunción (IICS-UNA) and the Hospital Pediátrico Niños de Acosta Ñu. Informed consent was obtained from all subjects involved in this study. The clinical investigation was conducted according to the principles expressed in the Declaration of Helsinki.

### Statistical analysis

The Chi-square test and Fisher’s exact test were used to compare categorical variables; continuous variables and non-normally distributed data were compared using the Mann-Whitney *U*-test; normally distributed data were compared using unpaired *t*-tests. Comparative analyses between experimental conditions (i.e., MOCK, IAV, SP or IAV + SP) were performed using one-way ANOVA with Tukey’s post-hoc test or Kruskal-Wallis analysis with Dunn’s post-hoc tests. Receiver operating curve (ROC) analysis was used to determine the optimal cut-off value for IP-10 to differentiate between non-severe and severe pneumonia cases. *P* < 0.05 was considered statistically significant. All statistical analyses were performed using GraphPad Prism (La Jolla, California, United States).

## Additional Information

**How to cite this article**: Hoffmann, J. *et al*. Viral and bacterial co-infection in severe pneumonia triggers innate immune responses and specifically enhances IP-10: a translational study. *Sci. Rep.* **6**, 38532; doi: 10.1038/srep38532 (2016).

**Publisher's note:** Springer Nature remains neutral with regard to jurisdictional claims in published maps and institutional affiliations.

## Supplementary Material

Supporting Information

## Figures and Tables

**Table 1:** 

Patient characteristics	NON-SEVERE PNEUMONIA n = 44		SEVERE PNEUMONIA n = 30		*P*
Age, months, median (IQR)	13.5	(6–26.7)	14	(8–28)	0.78
Weight, kg, median (IQR)	9.4	(7.5–12.2)	10.5	(8.3–12.5)	0.41
Gender, male	20	45.5	24	80.0	**<0.01**
***Clinical examination***					
Axillary temperature, °C, median (IQR)	37.6	(36.8–38.3)	37.9	(37–38)	0.56
Breathing rate, cycles/min., median (IQR)	49.5	(44.2–56.7)	56	(48.8–64.5)	**0.02**
Cardiac rate, cycles/min., median (IQR)	140.5	(127.8–152.8)	152.5	(139.5–160.5)	**0.01**
Neutrophils, %, median (IQR)	68	(56.5–78)	70	(54.5–78.5)	0.98
Oxygen saturation, %, median (IQR)	94	(92–97.7)	95	(90–96)	0.45
***Chest examination***					
Cough, n (%)	44	100	29	96.7	0.41
Dyspnea, n (%)	33	75.0	30	100.0	**<0.01**
Cyanosis, n (%)	2	4.5	7	23.3	**0.03**
Lower chest indrawing, n (%)	28	63.6	29	96.7	**<0.001**
Dullness to percussion, n (%)	3	6.8	12	40.0	**<0.001**
Pulmonary crackles, n (%)	31	70.5	27	90.0	0.05
Inability to drink, n (%)	3	6.8	5	16.7	0.26
Vomiting, n (%)	6	13.6	5	16.7	0.75
Prostration or lethargy, n (%)	0	0.0	18	60.0	**<0.0001**
Convulsions, n (%)	0	0.0	1	3.3	0.41
***Chest radiography interpretation***					
Pleural effusion with parenchymatous infiltrate, n (%)	4	9.1	15	50.0	**<0.001**
Pleural effusion without parenchymatous infiltrate, n (%)	1	2.3	3	10.0	0.30
Generalized dense homogenous opacification, n (%)	1	2.3	4	13.3	0.15
Localized dense homogenous opacification, n (%)	21	47.7	11	36.7	0.47
Interstitial syndrome, n (%)	19	43.2	8	26.7	0.22
Alveolar infiltrate, n (%)	6	13.6	1	3.3	0.23
Pneumothorax, n (%)	1	2.3	0	0.0	1.00
***Medical history***					
Heart condition, n (%)	6	13.6	0	0.0	0.08
Lung disease, n (%)	12	27.3	9	30.0	0.80
Tuberculosis, n (%)	1	2.3	1	3.3	1.00
Asthma, n (%)	1	2.3	1	3.3	1.00
Contracted ILI^a^, n (%)	14	31.8	15	50	0.15
Pneumococcal conjugate vaccine, n (%)	11	25.0	4	13.3	0.25
DPT–Hep. B–Hib vaccine: 1 dose, n (%)	28	63.6	22	73.3	0.45
DPT–Hep. B - Hib vaccine: 3 doses, n (%)	20	45.5	18	60.0	0.24
Influenza A/H1N1 vaccine	4	9.1	8	26.7	0.06
***Hospitalization follow-up***					
Pneumonia complication, n (%)	5	11.4	14	46.7	**<0.001**
Length of hospitalization, days, median (IQR)	3	(2–5)	7	(3.8–12.3)	**<0.001**
Death, n (%)	0	0.0	2	6.7	0.16

^a^Within the two previous weeks before study inclusion. Significant *P* values (<0.05) are in bold.

**Table 2:** 

Blood-based biomarkers	NON-SEVERE PNEUMONIA n = 44		SEVERE PNEUMONIA n = 30		*P*
CRP^a^	36	(12–96)	192	(24–192)	**0.01**
IP-10	2,090	(1,033–8,040)	8818	(1,384–43,886)	**<0.01**
PCT	0.3	(0.1–0.9)	1.46	(0.5–5.6)	**<0.01**
G-CSF	62.6	(0–833)	684.2	(0–4,209)	**0.03**
IFN-γ	84.9	(0–391.8)	149.8	(0–308)	0.69
IL-10	15.6	(0–72.4)	24.2	(0–87.1)	0.53
IL-12p70	211.1	(84.8–411)	305	(93.2–407.9)	0.31
IL-13	10.9	(5.4–20.4)	13	(8.3–31.5)	0.17
IL-1β	1.9	(0.3–6.9)	4.4	(1.1–8.9)	0.18
IL-1RA	472	(116.2–1,015)	466.6	(165.9–1,289)	0.47
IL-6	116.4	(41.1–376.5)	559.8	(139.9–2,129)	**<0.01**
IL-7	53	(31.1–84.1)	51.6	(17.3–76.0)	0.43
IL-8	49.1	(21.2–144.6)	117.2	(28.3–393.5)	**0.03**
IL-9	51.4	(21.5–92.5)	68.8	(24.4–97.6)	0.37
MCP-1	26	(0–203.8)	108.1	(0–672.7)	0.19
MIP-1β	169.6	(127.6–251.1)	260	(150.5–381.3)	**0.01**
PDGF-BB	16,773	(9,624–28,393)	14,111	(7,055–26,165)	0.29
RANTES	34,286	(24,326–48,594)	36,218	(25,565–53,832)	0.55
VEGF	1,741	(577.4–4,633)	3,100	(1,400–5,913)	0.11

^a^Only CRP data are expressed in mg L-1 otherwise in pg ml^−1^. Values are expressed as median (IQR) in pg ml^−1^. Differences between groups were compared using unpaired Mann-Whitney tests; significant changes (*P* *<* 0.05) are in bold.

**Table 3:** 

Group	Viral and bacterial associations	n	Description of viral and/or bacterial associations
**No etiology**	No pathogen detected	2	–
**Single detection**	**V**_(NA)_	7	(AdV), n = 1; (EV), n = 1; (HRV), n = 2; (RSV), n = 1; (PIV), n = 2
	**B**_(NA)_	3	(Sp_6AB), n = 2; (Sp_22F), n = 1
**Multiple detection of pathogen** (in nasal aspirates)	x**V**_(NA)_	1	(HCoV_NL63 + HBoV), n = 1
	x**B**_(NA)_	3	(Sp_3 + Sp_35 F), n = 1; (Sp_14 + Sp_19F), n = 1; (Sp_3 + Sa), n = 1
	**V**_(NA)_ + x**B**_(NA)_	10	(HRV + Sp_6AB + Sp_5), n = 1; (HMPV + Sp_9 V + Sp_10 A), n = 1; (EV + Sp_6AB + Sp_9V), n = 1; (HCoV_OC43 + Sp_7c + Sp_14), n = 1; (HRV + Mp + Sp_NT), n = 1; (RSV + Sa + Sp_19F), n = 1; (AdV + Hib + Sp_14), n = 1; (HRV + Sa + Sp_14), n = 3
	x**V**_(NA) +_ **B**_(NA)_	8	(HRV + AdV + Sa), n = 1; (RSV + IVB + Sp_NT), n = 1; (PIV-4 + HCoV_OC43 + Sp_NT), n = 1; (HMPV + EV + Sp_16F), n = 1;
			(AdV + HCoV_229E + Sp_14), n = 1; (HMPV + HCoV_229E + AdV + Sp_16 F), n = 1; (IAV 2009 pdm + RSV + Sp_19F), n = 1; (HBoV + HCoV_HKU + Sp_4), n = 1
	x**V**_(NA)_ + x**B**_(NA)_	4	(HBoV + IVB + PIV_1 + EV + AdV + Sp_6 A+ Sp_34), n = 1; (PIV_3 + HMPV + Sp_16 F + Sp_22F), n = 1; (IVB + PIV_3 + Sp_Sg18 + Sp_20 + Sp_35B), n = 1; (HPIV_4 + HRV + AdV + HiB + Sp_NT), n = 1
	**V**_(NA)_ + **B**_(NA)_	12	(HRV + Sp_NT), n = 1; (HRV + Sa), n = 1; (HRV + Sp_14), n = 2; (RSV + Sp_6AB), n = 3; (EV + Sp_19 A), n = 1; (RSV + Sp_23F), n = 1; (AdV + Sp_14), n = 2; (HCoV_OC43 + Sp_NT), n = 1
**Multiple detection of pathogen** (in nasal aspirates and blood samples)	**B**_(NA)_ + **B**_(BL)_	2	(Sp_14)_NA_ + (Sp_14)_BL_, n = 1; (Sa)_NA_ + (Sp_NT)_BL_, n = 1
	x**B**_(NA)_ + **B**_(BL)_	1	(Hib + Sp_9V)_NA_, n = 1 + (Sp_9V)_BL_, n = 1,
	**V**_(NA)_ +x**B**_(BL)_	2	(PIV_3)_NA_, n = 1 + (Sp_NT + Hib)_BL_, n = 2
	**V**_(NA)_ + **B**_(BL)_	1	(HRV)_NA_ + (Sp_20)_BL_, n = 1
	x**V**_(NA)_ + **B**_(BL)_	1	(RSV + HRV)_NA_ + (Hib)_BL_, n = 1
	**V**_(NA)_ + **B**_(NA)_ + **B**_(BL)_	7	(HRV)_NA_ + (Sp_NT)_NA_ + (Sp_NT)_BL_, n = 1; (HBoV)_NA_ + (HiB)_NA_ + (Hib)_BL_, n = 1; (HRV)_NA_ + (Sp_NT)_NA_ + (Sp_14)_BL_, n = 2;
			(PIV_3)_NA_ + (Sp_14)_NA_ + (Sp_14)_BL_, n = 1; (EV)_NA_ + (Sp_NT)_NA_ + (Sa)_BL_, n = 1; (HRV)_NA_ + (Sa)_NA_ + (Sp)_BL_, n = 1
	x**V**_(NA)_ + **B**_(NA)_ + **B**_(BL)_	3	(HBoV + RSV + PV)_NA_ + (Sp_6AB)_NA_ + (Sp_NT)_BL_; n = 1 ; (IVB + PIV_4)_NA_ + (Sa + Sp_NT)_NA_ + (Sp_NT)_BL_, n = 1; (HBoV + HCoV_HKU)_NA_ + (Sp_14)_NA_ + (Sp_NT)_BL_, n = 1
	x**V**_(NA)_ + x**B**_(NA)_ + **B**_(BL)_	2	(IVB + HRV)_NA_ + (Sp_14 + Sp_6AB)_NA_ + (Sp_14)_BL_, n = 1; (HRV + AdV)_NA_ + (Sa + Sp_6AB + Sp_8)_NA_ + (Hib)_BL_, n = 1
	**V**_(NA)_ + **B**_(NA)_ + x**B**_(BL)_	2	(RSV)_NA_ + (Sp_17 F)_NA_ + (Hib)_BL_, n = 1; (HRV)_NA_ + (Sp_7F)_NA_ + (Sp_NT)_BL_, n = 1
	**V**_(NA)_ + x**B**_(NA)_ + **B**_(BL)_	1	(HRV)_NA_ + (Sa + Sp_14)_NA_ + (Sp_14)_BL_, n = 1
	**V**_(PE)_ + **B**_(NA)_ + **B**_(BL)_	1	(HBoV)_PE_ + (Sp_3)_NA_ + (Sp_3)_PE_, n = 1
	**V**_(NA)_ + **B**_(NA)_ + **B**_(PE)_	1	(HRV)_NA_ + (Sp_14)_NA_ + (Sp_14)_PE_, n = 1
**TOTAL**		74	

V: Virus; B: Bacteria; NA: Nasal aspirate; BL: Blood; PE: Pleural effusion; x: several. AdV: Adenovirus; EV: Enterovirus; HRV: Human rhinovirus; RSV: Respiratory syncytial virus; PIV: Parainfluenza virus; HCoV: Human coronavirus; HBoV: Human bocavirus; HMPV: Human metapneumovirus; IAV 2009 pdm: 2009 pandemic Influenza virus A/H1N1; IVB: Influenza virus B; Sp: *Streptococcus pneumoniae*, serotype 6 AB, 6A, 3, 4, 34, Sg18, 20, 23 F, 17 F, 35B, 35 F, 14, 9 V, 19 F, 10 A, 7c, 16 F, 19 A; NT: non-typable; Sa: *Staphylococcus aureus*; Mp: *Mycoplasma pneumoniae*; Hib: *Haemophilus influenzae* type b.

**Table 4:** 

	NON-SEVERE PNEUMONIA (n = 44)				SEVERE PNEUMONIA (n = 30)				TOTAL (n = 74)				*P*
	n	(%)	IP-10	(IQR)	n	(%)	IP-10	(IQR)	n	(%)	IP-10	(IQR)	
***No respiratory pathogen detected***	2	(4.5)	652.1	(637.6–666.6)	0	(0.0)	0	0	2	(2.7)	652.1	(637.6–666.6)	—
***Single detection***	5	(11.4)	2,932	(903.8 – 10,388)	5	(16.7)	1,296	(938.8 – 1,307)	10	(13.5)	1,300	(974.9 – 4,237)	0.53
***Multiple detection of pathogen***	35	(79.5)	2,167	(1,173 – 7,953)	25	(83.3)	14,427	(3,981 – 82,994)	62	(83.8)	4,743	(1,445 – 14,570)	**<0.01**
**TOTAL**	44	(59.5)	2,090	(1,033 – 8,040)	30	(40.5)	8,818	(1,384 – 43,886)	74	(100.0)	3,312	(1,239 – 12,512)	**<0.01**

^a^IP-10 values are expressed in pg ml^-1^. IP-10 concentration differences between groups were compared using unpaired Mann-Whitney tests; significant changes (*P* < 0.05) are in bold.
