# Supplementary material for: Viral and bacterial co-infection in severe pneumonia triggers innate immune responses and specifically enhances IP-10: a translational study
Source: Sci Rep. 2016 Dec 6;6:38532. doi: 10.1038/srep38532 (PMC5138590; doi:10.1038/srep38532)
Supplement: Supporting Information [file srep38532-s1.pdf]

SUPPLEMENTARY INFORMATION

Viral and bacterial co-infection in severe pneumonia triggers innate immune responses and specifically enhances IP-10: a translational study. Jonathan Hoffmann[1], Daniela Machado[1], Olivier Terrier[2], Stephane Pouzol[1], Mélina Messaoudi[1], Wilma Basualdo[3], Emilio E Espínola[4], Rosa M. Guillen[4], Manuel Rosa-Calatrava[2], Valentina Picot[1], Thomas Bénet[5], Hubert Endtz[1], Graciela Russomando[4], Gláucia Paranhos-Baccalà[1*].

Figure S1

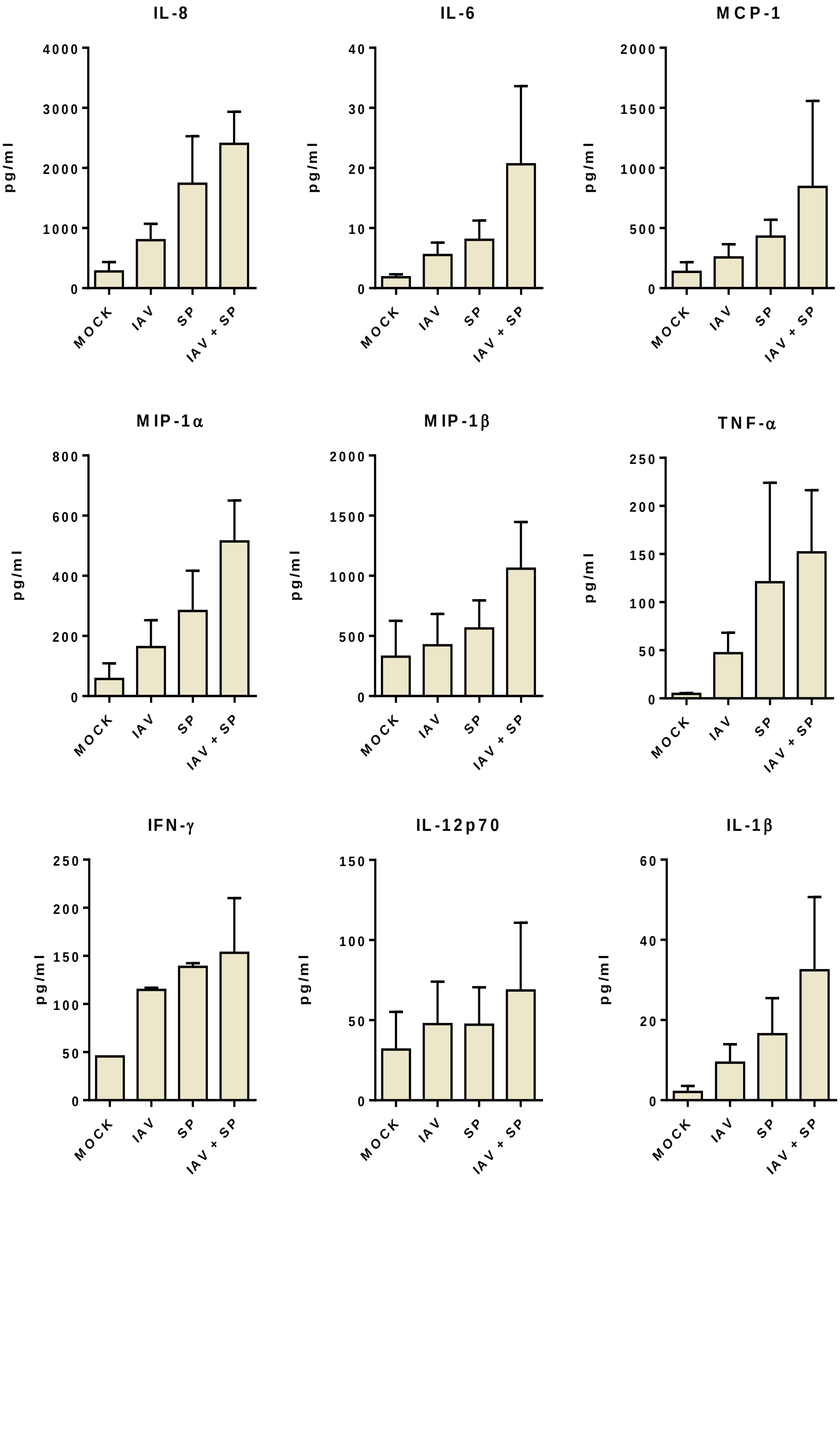
IL-8
IL-6
MCP-1
MIP-1α
MIP-1β
TNF-α
IFN-γ
IL-12p70
IL-1β
pg/ml
MOCK
IAV
SP
IAV + SP

<u>Figure S1 legend:</u> Cytokine and chemokine responses of MDMs after single or mixed infection with IAV and SP.

Preceding IAV infection leads to elevated secretion of pro-inflammatory cytokines and chemokines after co-infection with SP. MDMs were infected with or without IAV for 4 hours, then infected with or without SP, incubated for 20 h, and the concentrations of 27 cytokines and chemokines in the cell supernatants were measured using a quantitative multiplex immunoassay. A selection of deregulated expression profiles at 24 hours post-infection is shown: IP-10, IL-8, IL-6, MCP-1, MIP-1α, MIP-1β, TNF-α, IFN-γ and IL-12p70. Values are mean (± SEM) of three independent experiments. Statistical analyses were performed using one-way ANOVA with Tukey's post-hoc test.

Table S1: **Complete mRNA expression dataset of human monocyte-derived macrophages following single or mixed IAV and/or SP infection.**

Gene	Description	Fold Change (comparing to control group)		
		IAV	SP	IAV + SP
CXCL10	Chemokine (C-X-C motif) ligand 10	67,9	183,5	240,9
MX1	Myxovirus (influenza virus) resistance 1, interferon-inducible protein p78 (mouse)	134,0	120,3	151,4
IL1B	Interleukin 1, beta	0,6	61,4	59,4
IL23A	Interleukin 23, alpha subunit p19	3,3	68,1	56,2
CD80	CD80 molecule	2,4	49,5	54,3
STAT1	Signal transducer and activator of transcription 1, 91kDa	31,0	44,2	52,3
CCL2	Chemokine (C-C motif) ligand 2	2,2	26,9	34,6
DDX58	DEAD (Asp-Glu-Ala-Asp) box polypeptide 58	11,2	19,3	22,2
STAT4	Signal transducer and activator of transcription 4	1,7	16,4	15,8
IL10	Interleukin 10	1,3	11,6	10,6
IRF7	Interferon regulatory factor 7	6,6	6,1	6,8
CSF2	Colony stimulating factor 2 (granulocyte-macrophage)	0,7	5,7	6,3
IL8	Interleukin 8	0,1	7,1	6,2
LYZ	Lysozyme	12,0	6,6	5,5
IFNB1	Interferon, beta 1, fibroblast	2,3	5,1	5,2
ICAM1	Intercellular adhesion molecule 1	0,5	5,0	4,7
CD40	CD40 molecule, TNF receptor superfamily member 5	0,9	4,4	4,7
IL6	Interleukin 6 (interferon, beta 2)	0,7	3,7	4,0
TNF	Tumor necrosis factor	0,5	3,7	3,7
IL1R1	Interleukin 1 receptor, type I	0,4	3,9	3,5
NFKB1	Nuclear factor of kappa light polypeptide gene enhancer in B-cells 1	1,1	3,4	3,4
CCL5	Chemokine (C-C motif) ligand 5	0,8	3,5	3,4
NFKBIA	Nuclear factor of kappa light polypeptide gene enhancer in B-cells inhibitor, alpha	0,4	3,4	3,2
CCR4	Chemokine (C-C motif) receptor 4	1,1	2,5	3,1
MYD88	Myeloid differentiation primary response gene (88)	2,1	2,3	2,9
TLR8	Toll-like receptor 8	0,8	2,4	2,4
TLR9	Toll-like receptor 9	3,9	2,4	2,1
IL1A	Interleukin 1, alpha	0,5	3,7	2,1
FOXP3	Forkhead box P3	1,8	2,4	2,1
CASP1	Caspase 1, apoptosis-related cysteine peptidase (interleukin 1, beta, convertase)	1,5	2,0	2,0
TYK2	Tyrosine kinase 2	1,3	2,0	1,9
TICAM1	Toll-like receptor adaptor molecule 1	1,2	2,2	1,9
NOD2	Nucleotide-binding oligomerization domain containing 2	1,4	1,8	1,8
IRF3	Interferon regulatory factor 3	1,3	1,6	1,8
HLA-A	Major histocompatibility complex, class I, A	1,5	1,6	1,7
TLR2	Toll-like receptor 2	1,2	1,5	1,5
STAT6	Signal transducer and activator of transcription 6, interleukin-4 induced	1,6	1,4	1,5
IL5	Interleukin 5 (colony-stimulating factor, eosinophil)	0,7	1,1	1,4
STAT3	Signal transducer and activator of transcription 3 (acute-phase response factor)	1,1	1,3	1,3
NLRP3	NLR family, pyrin domain containing 3	1,1	1,2	1,3
IFNAR1	Interferon (alpha, beta and omega) receptor 1	0,6	1,4	1,2
HLA-E	Major histocompatibility complex, class I, E	1,3	1,1	1,2
CXCR3	Chemokine (C-X-C motif) receptor 3	0,6	1,4	1,2
NOD1	Nucleotide-binding oligomerization domain containing 1	1,4	1,0	1,1
CD14	CD14 molecule	0,9	1,2	1,1
IL18	Interleukin 18 (interferon-gamma-inducing factor)	0,9	1,2	1,1
APCS	Amyloid P component, serum	0,7	1,1	1,0
CCR6	Chemokine (C-C motif) receptor 6	0,7	1,1	1,0
CCR8	Chemokine (C-C motif) receptor 8	0,7	1,1	1,0
CD40LG	CD40 ligand	0,7	1,1	1,0
CD8A	CD8a molecule	0,7	1,1	1,0
CRP	C-reactive protein, pentraxin-related	0,7	1,1	1,0
FASLG	Fas ligand (TNF superfamily, member 6)	0,7	1,1	1,0
GATA3	GATA binding protein 3	0,7	1,1	1,0
IFNA1	Interferon, alpha 1	0,7	1,1	1,0
IFNG	Interferon, gamma	0,7	1,1	1,0
IL13	Interleukin 13	0,7	1,1	1,0
IL17A	Interleukin 17A	0,7	1,1	1,0
IL2	Interleukin 2	0,7	1,1	1,0
IL4	Interleukin 4	0,7	1,1	1,0
MBL2	Mannose-binding lectin (protein C) 2, soluble	0,7	1,1	1,0
MPO	Myeloperoxidase	1,7	1,1	1,0
RORC	RAR-related orphan receptor C	0,7	1,1	1,0
TBX21	T-box 21	0,7	1,1	1,0
TLR3	Toll-like receptor 3	1,1	0,8	1,0
CD4	CD4 molecule	1,6	1,1	1,0
TLR5	Toll-like receptor 5	1,3	1,1	1,0
CCR5	Chemokine (C-C motif) receptor 5	4,4	1,1	1,0
CD86	CD86 molecule	1,2	0,9	1,0
LY96	Lymphocyte antigen 96	0,9	1,0	1,0
C3	Complement component 3	0,7	1,0	0,9
IRAK1	Interleukin-1 receptor-associated kinase 1	1,2	0,9	0,9
MAPK1	Mitogen-activated protein kinase 1	0,9	1,1	0,9
TRAF6	TNF receptor-associated factor 6	1,0	1,0	0,9
JAK2	Janus kinase 2	0,8	0,7	0,7
TLR1	Toll-like receptor 1	0,6	0,6	0,7
MAPK8	Mitogen-activated protein kinase 8	0,7	0,8	0,7
IFNGR1	Interferon gamma receptor 1	0,7	0,6	0,5
RAG1	Recombination activating gene 1	0,7	0,4	0,5
SLC11A1	Solute carrier family 11 (proton-coupled divalent metal ion transporters), member 1	1,5	0,4	0,4
TLR6	Toll-like receptor 6	0,5	0,4	0,4
TLR4	Toll-like receptor 4	0,8	0,4	0,4
ITGAM	Integrin, alpha M (complement component 3 receptor 3 subunit)	0,5	0,3	0,2
TLR7	Toll-like receptor 7	0,7	0,2	0,2

<u>Table S2</u>: Complete microRNA expression dataset of human monocyte-derived macrophages following single or mixed IAV and/or SP infection.

Human microRNA	Fold Change (comparing to control group)		
	IAV	SP	IAV + SP
hsa-miR-200a-3p	6,9	3,7	**7,3**
hsa-miR-298	3,3	1,1	**4,8**
hsa-miR-130a-3p	5,4	1,9	**4,8**
hsa-miR-20b-5p	1,9	4,9	**4,7**
hsa-miR-196a-5p	2,0	2,3	**4,3**
hsa-miR-147a	2,3	2,0	**4,1**
hsa-miR-138-5p	2,6	2,7	**3,9**
hsa-miR-135a-5p	1,4	2,0	**3,8**
hsa-let-7c-5p	1,2	2,9	**3,7**
hsa-miR-194-5p	2,8	1,2	**3,7**
hsa-miR-451a	1,9	3,1	**3,6**
hsa-miR-205-5p	4,1	0,5	**3,1**
hsa-miR-183-5p	3,0	1,8	**2,8**
hsa-miR-19b-3p	1,6	2,1	**2,6**
hsa-miR-195-5p	1,4	1,9	**2,6**
hsa-miR-17-5p	1,4	2,2	**2,5**
hsa-let-7e-5p	1,2	1,8	**2,5**
hsa-miR-16-5p	1,7	1,5	**2,4**
hsa-miR-20a-5p	1,3	2,3	**2,3**
hsa-miR-31-5p	2,1	2,3	**2,3**
hsa-miR-182-5p	1,9	1,7	**2,2**
hsa-miR-155-5p	3,0	1,3	**2,2**
hsa-miR-142-3p	1,4	2,3	**2,1**
hsa-miR-29c-3p	1,3	2,1	**2,1**
hsa-miR-18a-5p	1,1	1,9	**2,1**
hsa-let-7a-5p	1,1	1,4	**2,1**
hsa-let-7d-5p	1,0	1,8	**2,0**
hsa-miR-19a-3p	1,4	1,6	**2,0**
hsa-miR-30b-5p	1,3	1,6	**2,0**
hsa-miR-34a-5p	1,2	2,3	**1,9**
hsa-miR-363-3p	0,9	1,7	1,9
hsa-miR-148a-3p	1,4	1,0	1,9
hsa-miR-186-5p	1,1	1,4	1,9
hsa-miR-149-5p	1,7	0,9	1,8
hsa-miR-135b-5p	3,6	2,1	1,8
hsa-miR-181a-5p	0,9	2,0	1,8
hsa-let-7g-5p	1,6	1,4	1,8
hsa-miR-126-3p	1,2	1,3	1,7
hsa-miR-21-5p	2,0	1,2	1,7
hsa-miR-210-3p	0,9	1,1	1,7
hsa-miR-29b-3p	1,0	2,1	1,7
hsa-miR-98-5p	1,6	1,3	1,7
hsa-miR-26b-5p	1,5	1,3	1,7
hsa-miR-27b-3p	1,4	1,3	1,6
hsa-miR-27a-3p	1,0	1,4	1,6
hsa-miR-379-5p	1,7	0,7	1,6
hsa-miR-15a-5p	0,9	1,9	1,5
hsa-miR-128-3p	1,2	1,6	1,5
hsa-miR-15b-5p	1,3	1,5	1,5
hsa-miR-142-5p	1,0	2,8	1,5
hsa-miR-146a-5p	1,5	1,4	1,5
hsa-miR-335-5p	2,4	0,5	1,5
hsa-miR-103a-3p	1,2	1,5	1,4
hsa-miR-30e-5p	1,0	1,3	1,4
hsa-miR-134-5p	0,8	1,1	1,4
hsa-miR-152-3p	1,2	1,0	1,4
hsa-miR-409-3p	2,0	0,5	1,4
hsa-miR-223-3p	1,6	1,3	1,3
hsa-miR-9-5p	1,5	0,8	1,3
hsa-miR-214-3p	0,4	1,7	1,3
hsa-miR-99b-5p	1,3	1,0	1,2
hsa-miR-493-3p	1,0	1,5	1,2
hsa-miR-145-5p	1,0	1,7	1,2
hsa-miR-574-3p	1,1	1,1	1,2
hsa-miR-185-5p	1,3	1,1	1,2
hsa-miR-18b-5p	1,2	1,0	1,1
hsa-miR-143-3p	1,0	1,4	1,1
hsa-miR-23b-3p	1,3	1,1	1,1
hsa-miR-30c-5p	1,2	0,9	1,1
hsa-miR-187-3p	0,9	0,9	1,1
hsa-miR-191-5p	1,0	1,1	1,0
hsa-miR-132-3p	1,1	0,9	1,0
hsa-miR-383-5p	1,7	0,5	0,9
hsa-miR-26a-5p	1,2	1,1	0,9
hsa-miR-125a-5p	1,2	0,9	0,9
hsa-miR-299-3p	1,7	0,5	0,9
hsa-miR-105-5p	1,7	1,0	0,8
hsa-miR-302a-3p	0,8	1,5	0,8
hsa-miR-125b-5p	0,8	1,2	0,7
hsa-miR-325	1,3	0,6	0,6
hsa-miR-150-5p	1,7	0,5	0,5
hsa-miR-184	1,7	0,5	0,5
hsa-miR-203a-3p	1,7	0,5	0,5
hsa-miR-206	1,7	0,5	0,5

<u>Table S3:</u> Diagnostic accuracy values of the main significant blood-based biomarkers allowing differentiation between non-severe of severe pneumonia cases.

Serum-based biomarker	AUC	SE	[95% CI]	*P*	Cut-off value	Sen. (%)	Spe. (%)	LR
**PCT**	0.70	0.06	[0.58 - 0.82]	**<0.01**	>0.68	70.0	70.4	2.37
**IP-10**	0.69	0.06	[0.57 - 0.82]	**<0.01**	> 4,240	63.3	63.6	1.74
**IL-6**	0.70	0.07	[0.58 - 0.83]	**<0.01**	> 250.7	66.6	65.9	1.96
**CRP**	0.67	0.07	[0.54 - 0.79]	*.02*	> 72.00	70.0	61.3	1.81
**IL-8**	0.65	0.07	[0.51 - 0.78]	*.03*	> 88.70	63.3	63.6	1.74

AUC: area under curve; SE: standard error; CI: confidence interval; Sen.: Sensitivity; Spe.: Specificity; LR: likelihood ratio. Significant *P*-values ($P < 0.05$) are in bold.
